# Future Changes in Simulated Evapotranspiration across Continental Africa Based on CMIP6 CNRM-CM6

**DOI:** 10.3390/ijerph18136760

**Published:** 2021-06-23

**Authors:** Isaac Kwesi Nooni, Daniel Fiifi T. Hagan, Guojie Wang, Waheed Ullah, Jiao Lu, Shijie Li, Mawuli Dzakpasu, Nana Agyemang Prempeh, Kenny T. C. Lim Kam Sian

**Affiliations:** 1 Binjiang College, Nanjing University of Information Science & Technology, Wuxi 214105, China; nooni25593@alumni.itc.nl (I.K.N.); kennlks@gmail.com (K.T.C.L.K.S.); 2 Collaborative Innovation Center on Forecast and Evaluation of Meteorological Disasters, School of Geographical Sciences, Nanjing University of Information Science & Technology, Nanjing 210044, China; dans7messiah@nuist.edu.cn (D.F.T.H.); waheed.wama@nuist.edu.cn (W.U.); jiao_lu@nuist.edu.cn (J.L.); lishijie@nuist.edu.cn (S.L.); 3 Key Lab of Northwest Water Resources, Environment and Ecology, School of Environmental and Municipal Engineering, Xi’an University of Architecture and Technology, Xi’an 710055, China; mawuli.dzakpasu@xauat.edu.cn; 4 School of Geosciences, Department of Geographic Sciences, University of Energy and Natural Resources, Sunyani P.O. Box 214, Ghana; agyemang.prempeh@uenr.edu.gh; 5 Key Laboratory of Meteorological Disaster, Ministry of Education (KLME)/Joint International Research Laboratory of Climate and Environment Change (ILCEC), Collaborative Innovation Center on Forecast and Evaluation of Meteorological Disasters (CIC-FEMD), Nanjing University of Information Science and Technology, Nanjing 210044, China

**Keywords:** CMIP6, global climate model (GCM), CNRM-CM, SSP-RCPs, historical, projection, ET, Africa

## Abstract

The main goal of this study was to assess the interannual variations and spatial patterns of projected changes in simulated evapotranspiration (ET) in the 21st century over continental Africa based on the latest Shared Socioeconomic Pathways and the Representative Concentration Pathways (SSP1-2.6, SSP2-4.5, SSP3-7.0, and SSP5-8.5) provided by the France Centre National de Recherches Météorologiques (CNRM-CM) model in the Sixth Phase of Coupled Model Intercomparison Project (CMIP6) framework. The projected spatial and temporal changes were computed for three time slices: 2020–2039 (near future), 2040–2069 (mid-century), and 2080–2099 (end-of-the-century), relative to the baseline period (1995–2014). The results show that the spatial pattern of the projected ET was not uniform and varied across the climate region and under the SSP-RCPs scenarios. Although the trends varied, they were statistically significant for all SSP-RCPs. The SSP5-8.5 and SSP3-7.0 projected higher ET seasonality than SSP1-2.6 and SSP2-4.5. In general, we suggest the need for modelers and forecasters to pay more attention to changes in the simulated ET and their impact on extreme events. The findings provide useful information for water resources managers to develop specific measures to mitigate extreme events in the regions most affected by possible changes in the region’s climate. However, readers are advised to treat the results with caution as they are based on a single GCM model. Further research on multi-model ensembles (as more models’ outputs become available) and possible key drivers may provide additional information on CMIP6 ET projections in the region.

## 1. Introduction

Evapotranspiration (ET) is an important parameter of the water, carbon, and energy cycle [1,2,3,4,5]. Evaluating ET change is pivotal for formulating evidence-based strategies to enhance regional monitoring of water resource management and the design of policies that promote sustainable agriculture, mostly in water-limited regions in a warming climate [6,7,8]. Moreover, our current climate models show a rising temperature (T) expected to impact the hydrological cycle, thus increasing ET. Increases in ET are predicted to result in more frequent and intense extreme events (e.g., floods, droughts, and heatwaves) [9,10]. Thus, understanding our future climate under the current global warming would improve our response to the resulting impacts [6].

To project the future climate, global climate models (GCMs) are used [10,11,12,13,14,15,16]. Datasets from different climate centers provide opportunities for projecting simulations of climate variables [10,13,14]. Now, historical and simulated data records covering a longer period of time have been generated and are available for research [13,14]. The Sixth Phase of Coupled Model Intercomparison Project (CMIP6) is an updated version of phase 5 (CMIP5) with similar radiative forcing levels for 2100. The Representative Concentration Pathways (RCP2.6, RCP4.5, RCP6.0, and RCP8.5) are now known as the Shared Socioeconomic Pathways (SSP1-2.6, SSP2-4.5, SSP4-6.0, and SSP5-8.5) [12,17]. We refer readers to the following literature for more details on: CMIP-1 [13,18,19]; CMIP-2 [14,16], CMIP-3 [18,19,20,21], CMIP5 [20,22,23,24], and CMIP6 [25,26,27,28]:

Much of the literature on the projection of climate models has been conducted at the global and regional scales where GCM datasets are available [10,15]; however, they offer limited information on few variables of interest (such as ET) in regions with heterogeneity in biophysical and environmental conditions [29]. Indeed, precipitation (P) and T projections studies have been discussed in the literature under different CMIP frameworks. Even now, with CMIP6, there is sufficient evidence to suggest the occurrence of a simulated mean warming climate due to a rising T [12,19,20,21,24,27,30,31,32,33,34,35,36]. A warming climate certainly impacts P distribution, which influences ET in both space and time [10,27,28]. Moreover, this comes as a natural question in the study of projections of future changes in ET seasonality vis-a-vis recent evidence of significant changes in P and T in some parts of the world [22,25,27,30,31,32,37,38].

In the CMIP suite, the simulated ET variable comprises three components: plant transpiration, soil evaporation, and canopy interception [1,4]. Understanding ET interannual variations, spatial patterns, and how ET is represented in CMIP6 model simulations of the present and future climate may increase our confidence in the results for hydro-meteorological applications.

The CMIP framework is a community research tool developed to help us understand the physical climate response to future emissions in a warming climate [11,12,14,16]. According to Eyring et al. [12], advances in climate modeling technology coupled with enhanced resolutions have improved how we represent essential indicators of climate variables. For example, the CMIP6 archive has so far proven to have the advanced skill to capture large-scale patterns of climate variables [39] and would support climate change research in the upcoming years [40,41].

Unlike in previous studies where multi-models were evaluated and projected, we selected one model following the work of [42,43,44,45,46] but focusing only on future changes of ET as the variable of interest. In particular, we focused on CNRM-CM models from the France Centre National de Recherches Météorologiques. Our choice of CNRM-CM was dictated by data availability and previous evaluation studies of P and T [27]. We do not wish to describe all model components here, and their couplings are provided in Voldoire et al. with a detailed explanation [46]. Additionally, Voldoire et al. [46,47] provide a difference between CNRM-CM5 and CM6. We highlight a few of the remarkable changes or modifications made in the land surface model [48], atmospheric model [49], and ocean (sea-ice) model [50] component resolutions, respectively. The major upgrades and modifications improved the simulation outputs of tropical climates [46].

Africa is considered a hotspot of global warming (see AR4 and 5 [10,15] for detailed analyses). According to the authors of [10], the continent is warming, and a statistically significant warming trend is projected for Africa in the 21st century. A similar study is consistent with P projection over Africa. However, the projected ET changes are less clear. Africa is characterized by several climate zones based on the Köppen–Geiger climate classifications [51]. Across the continent, the hotspots of climate characteristics, such as land–atmosphere fluxes or near-surface temperature, can be found in some of these climate zones (e.g., arid and semi-arid regions), where P is closely dependent on soil moisture availability [52,53], making the continent a suitable regional test bed for studies. In this study, we can fairly assess the added benefits of the CNRM-CM6 model product without the added noise of the global scale [6,54]. We extend and build on previous studies to show a comprehensive picture of the future changes in ET over Africa under rapid warming and investigate their possible trends. Thus, following the works of [43,44,55] (using a single model) and [27] (i.e., climatology studies), the main aim of this research was to study future ET seasonality at regional scales, and the outcome from this study is highly important and relevant within the ongoing studies in climate change [10,15].

To study CMIP6 ET projections under the four combined scenarios of the SSP1-2.6, SSP2-4.5, SSP3-7.0, and SSP5-8.5 can provide insights into the regional development of future climate change policy [10].

The objectives for this study are (1) to examine the projected changes in simulated ET over Africa using the latest CNRM-CM6 of the CMIP6 dataset and (2) to quantify and analyze the interannual variations of projected ET seasonality across the different SSP-RCPs over Africa.

The rest of the paper is organized as follows. In Section 2, the data used and methods of study are described. In Section 3, the results are presented, and in Section 4, the discussion of the results is presented. The main conclusions of the study are summarized in Section 5.

## 2. Materials and Methods

### 2.1. Description of Study Area

Continental Africa is a climatologically diverse region located geographically between 32°00′ N and 35°00′ S and 14°00′ W and 52°00′ E. The continent has a huge landmass area stretching to nearly 30.37 million km^2^. The continent straddles the equator and is boarded to the southeast by the Indian Ocean, to the northeast by the Mediterranean Sea, and to the west by the Atlantic Ocean [56].

The observed mean P in Africa does not exceed 700 mm year^−1^, and the mean T ranges from 15 °C to 27 °C. The P and T distribution helps define African’s two distinct weather patterns, where the P distribution in space and time is modulated by the oscillation of the intertropical convergence zone (ITCZ) [57].

**Figure 1:**
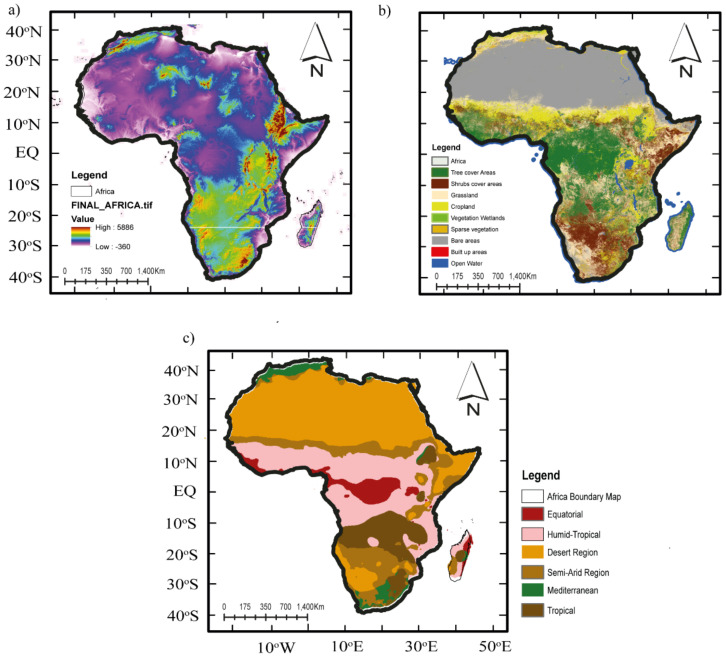
Geo-information of (**a**) digital elevation model (DEM) from NASA SRTM [58], (**b**) LULC from ESA CCI [63], and (**c**) climate zones from the Köppen climate map across Africa.

The presence of the huge oceans partly interacts with the global climate forcing systems, such as the El Niño–Southern Oscillation (ENSO) and the Indian Ocean Dipole (IOD). These teleconnections and the complex topography (Figure 1a) [58] partly help to explain the seasonality of different climatic variables [21,59].

The vegetation cover interacts in complex ways to affect weather and climate [60,61]. The continent has experienced years of land use land cover (LULC) change [62]. The period 1980–2005 reflects and captures all forms of anthropogenic activities in the region, particularly in global warming [61,62]. Figure 1b shows the 2016 LULC map from the European Space Agency Climate Change Initiative (ESA CCI) product [63]. Here, we regrouped the LULC into seven dominant classes: forest, shrubland, savannas, grassland, cropland, urban and built-up, and barren or sparsely vegetated. According to the authors of [61,62], widespread degradation is set to intensify due to unmanaged human activities (e.g., overgrazing, agricultural expansion, overexploitation, and deforestation) over some parts of the African region.

Figure 1c shows the Köppen climate map of the study area. In addition, Africa’s climate may be re-classified into seven different zones according to the Köppen–Geiger Classification (Figure 1c): arid (desert), semi-arid (Sahelian), humid-tropical, tropical, and Mediterranean [57]. Ongoing global warming has led to changes in the different climate factors such as P and ET. Knowledge about these changes and the response of these climate factors (e.g., ET) to global warming and their implications for nature and human society is valuable [3,52].

### 2.2. Climate Modeling Data

To highlight the spatial pattern and interannual variabilities of simulated ET, we focused our analyses fully on the CNRM-CM6 future projections (2015–2100). The GCM CNRM-CM6 outputs and statistical procedures are discussed in the subsections below. The global monthly CNRM-CM6 product used in this study is briefly described below.

The variable used in this study was ET (denoted as evspsbl) in the CMIP6 archive at a spatial resolution of 1.4° × 1.4°, and we used one ensemble member (r1i1p1;) for this analysis, where (r1) denotes the first initial conditions for the first initialization method (i1) using the first set of physics (p1). The CNRM-CM-6 output ran under an updated form of RCP, that is, SSP1-2.6, SSP2-4.5, SSP4-6.0, and SSP5-8.5. The CNRM-CM of the CMIP model was chosen based on the data availability, and the CM6 has historical data (from 1850 to 2014 and from 2015 to 2100). We used the monthly outputs of the CNRM-CM6 climate model data to examine the projection of simulated ET.

### 2.3. Statistical Analysis of Historical and Projection of ET

Different statistical methods were used to achieve the objectives of this study. First, we performed unit conversion where necessary. The ET products were converted from their respective units (kilograms per square meter per second) to millimeters per month, accounting for the number of days each month.

Second, the GCMs CNRM-CM6 dataset was aggregated to the annual and seasonal timescale. We computed and quantified seasonal climatology for the entire continental Africa by spatially averaging the long-term ET over the study area. We selected the period 1995–2014 as our baseline period for historical analysis. The unit of analysis used for the projection was annual and seasonal changes. The projected changes in ET for the SSP-RCPs scenarios were based on three time slices: near future (2020–2035), mid-century (2050–2069), and end-of-the-century (2080–2099) [64]. These time slices provide information on the expected magnitude of the climate response over each time window [50]. In addition, we used time series analysis to analyze temporal variation in the historical observations and projections of simulated ET in CNRM-CM6. Further, to determine the projected climate change signals for each time window, we calculated the difference between the future time window and the reference period (i.e., 1995–2014) and projected from 2015 to 2100 under the SSP-RCPs (SSP1-2.6, SSP2-4.5, SSP3-7.0, and SSP5-8.5) emission scenarios.

Finally, we applied the linear trend analysis to estimate the projected ET trends over the region. Simultaneously, the significance of these linear trends was examined by Mann–Kendall trend analysis [65,66,67]. For the detailed computation procedure, please refer to the studies of [68,69,70].

## 3. Results

This section describes the simulated ET climatology (i.e., annual and seasonal mean changes) across Africa. We focused on the spatial pattern and interannual variations of the historical and SSP-RCPs (i.e., SSP1-2.6, SSP2-4.5, SSP3-7.0, and SSP5-8.5).

### 3.1. Projected Changes in Annual and Seasonal ET

#### 3.1.1. Temporal Variations

**Figure 2:**
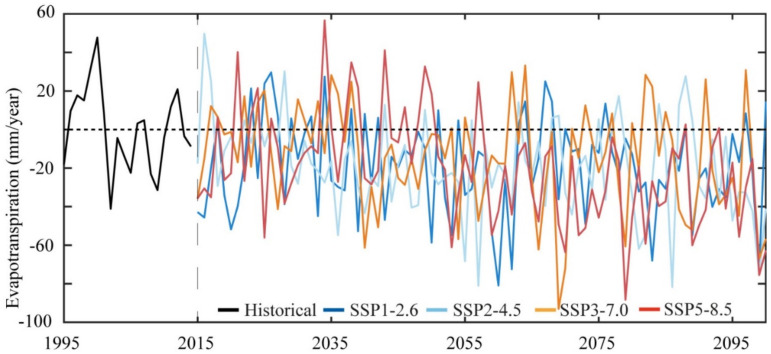
The annual ET in the historical period (1995–2014) and future period (2015–2100) under SSP1-2.6, 2-4.5, 3-7.0, and 5-8.5 scenarios relative to the historical mean.

Figure 2 depicts the interannual variations of ET for historical and future periods over Africa from 1995 to 2100 under the new SSP-RCPs scenarios (i.e., SSP1-2.6, SSP2-4.5, SSP3-7.0, and SSP5-8.5) relative to the historical mean.

Generally, the results showed that ET during the historical period showed different variations from 1995 to 2014, but it showed a decreasing rate up to 5 mm year^−1^ at the end of the present-day climatology (Figure 2, black line). Under SSP1-2.6 and SSP5-8.5, ET was projected to decrease from 2015 to 2019, and then recover (significantly increasing from 2000 to 2035, before decreasing from 2036 to 2100). We projected a prominent recovery of ET by a magnitude ranging 0–20 mm year^−1^ under the SSP1-2.6 at the beginning of the near future (NF) (2020–2035), slightly decreasing after 2035 at a magnitude range of 5–60 mm year^−1^, especially at the beginning of the NF, before slightly decreasing at the end of 2035. The simulated ET was projected to remain negative through the mid-century (MC) (i.e., 2055) until the end of the century (EC) (2100).

A similar magnitude was projected under SSP2-4.5, where ET increased shortly after 2015, and was then projected to decrease at the end of 2100, albeit with intermittent increases disappearing from 2035 to 2100. The decreasing ET trend from 2015 to 2100 was shown under SSP3-7.0 and SSP5-8.5, with subsequent recovery of a slightly increasing trend and decreasing afterward over the projected future period.

However, under SSP3-7.0, the study projected a relatively small increase (up to a few years) compared to the other remaining scenarios. The SSP1-2.6 and SSP5-8.5 showed the highest projected ET decrease compared to the other two SSPs. Based on the above results, this study found complex changes in the simulated ET during 2015–2100, with the specific results of an increase (decrease) first, followed by a decrease (increase), and, finally, a return to an increase (decrease) for the historical and future periods.

**Figure 3:**
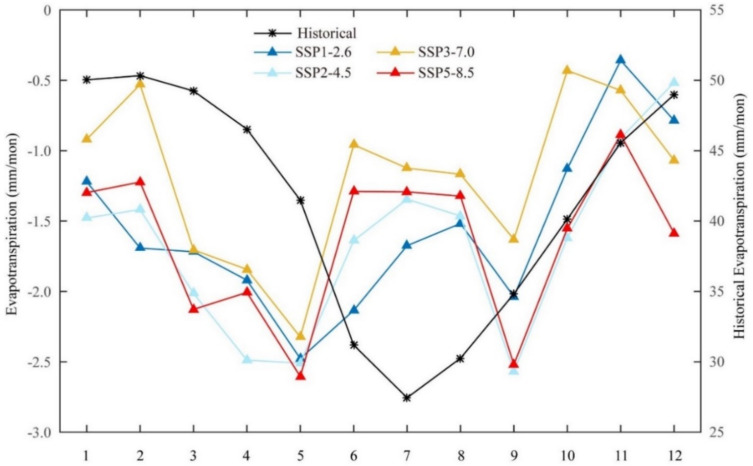
Seasonal variations of ET in the historical period (1995–2014) and future period (2015–2100) under the SSP1-2.6, 2-4.5, 3-7.0, and 5-8.5 scenarios. (Note: the historical period in the figure is the original value, using the right coordinate; the future period under four scenarios is computed by subtracting the historical mean from the entire time series, using the left coordinate). The unit is mm month^−1^.

Figure 3 shows the seasonal variations of ET based on the historical mean (1995–2014, black line) under the SSP-RCPS (i.e., SSP1-2.6, SSP2-4.5, SSP3-7.0, and SSP5-8.5) scenarios (colored lines). The historical monthly simulated ET climatology was computed for 1995–2014 to explain the seasonal variability. The result presents strong temporal variations (in historical ET, black color) and ranges from 27.5 to 50.1 mm mon^−1^. We observed ET seasonality starting from boreal winter and slightly declining in boreal spring. For example, the peak in February was recorded as 50.1 mm mon^−1^ and then slightly decreased in March (49.5 mm mon^−1^) and April (46 mm mon^−1^). On the other hand, we observed boreal summer as having the lowest in ET seasonality. June had the record with 31 mm mon^−1^ and August with 30.1 mm mon^−1^, while July had the lowest at 27.5 mm mon^−1^.

Figure 3 shows the monthly ET changes in the future climate (i.e., relative to the baseline period) (Figure 3: SSP1-2.6, SSP2-4.5, SSP3-7.0, SSP5-8.5). For the projections, the projected monthly ET climatology was computed by subtracting the historical mean from the entire time series for the SSP1-2.6, 2-4.5, 3-7.0, and 5-8.5 scenarios.

The future seasonal climatology projected a different pattern relative to the historical one (Figure 3). The results projected a temporal trend ranging from 0.5 to 3 mm mon^−1^. The ET changed peak in February, June, and October for all scenarios (Figure 3). The seasonal pattern of projected ET in all the SSP-RCPs showed that the magnitude of change was higher in October–November (OND) than in June–August (JJA) and in February. SSP1-2.6 and SSP2-4.5 projected a lower seasonal mean than the SSP3-7.0 and SSP5-8.5 scenarios. SSP1-2.6 and SSP2-4.5 projected a decrease from winter to summer, but ET increased in autumn relative to the SSP3-7.0 and SSP5-8.5 scenarios.

#### 3.1.2. Spatial Variations

**Figure 4:**
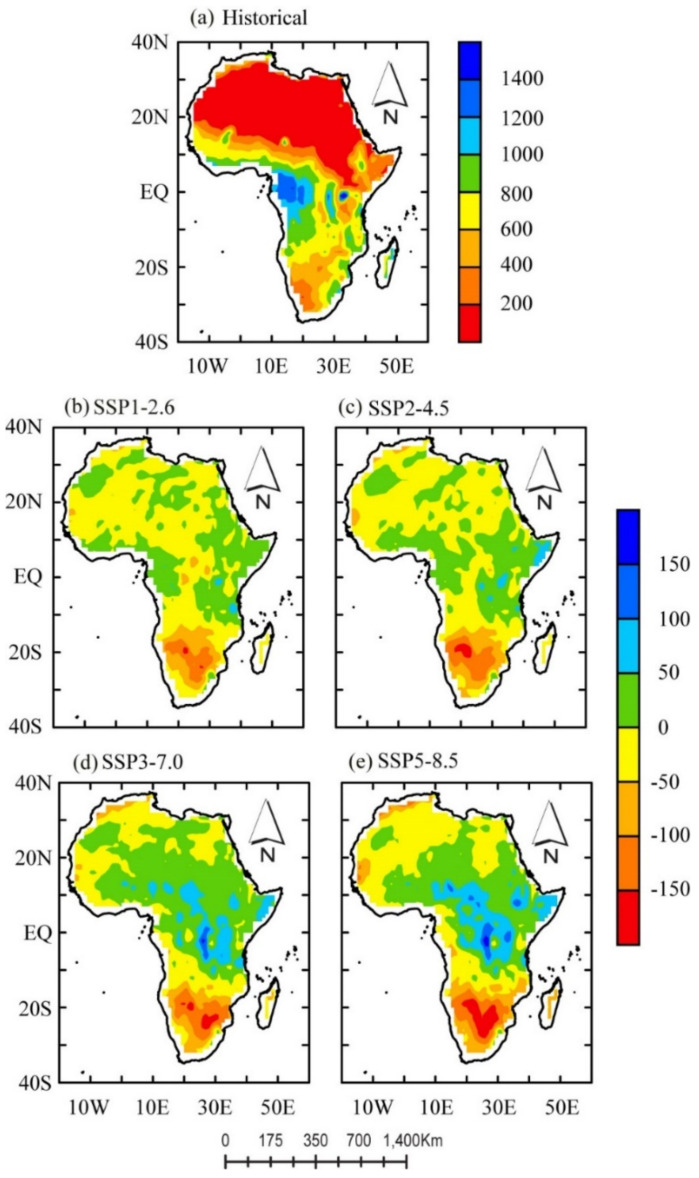
Spatial distribution of multi-year mean evapotranspiration (**a**) in the historical period (1995–2014) and (**b**–**e**) ET change in the future period (2020–2099) under the SSP1-2.6, 2-4.5, 3-7.0, and 5-8.5 scenarios relative to the historical mean. The unit is mm year^−1^.

To better understand the ET seasonality and quantify their relative spatial distribution in the future climate across the continent, we calculated and compared the spatial distribution of the multi-year mean ET in the historical (1995–2014) and future (2020–2100) periods. Figure 4 depicts the spatial land ET distribution associated with the variations of the historical and all SSP-RCPs scenarios.

In Figure 4a, we present the spatial distribution of the multi-year mean ET in the historical periods (1995–2014). We observed a spatially distinct band of average annual historical ET (Figure 4a) distributed in the humid tropical (i.e., along the west of the Sahelian belt stretching to the Guinean coast). The average annual ET with the highest value was distributed in equatorial regions at 5° S–5° N (i.e., the interior of the Congo Forest), while it was lowest in the Sahara Desert (in the geographic locations of 20° N–35° N and small patches of arid zones in the east of the Sahelian belt to the Horn of Africa (HoA) and the Kalahari Desert in the south).

The annual ET presented a spatially distinct band of ET distribution in the range of <200 to >1000 mm (Figure 4a). The highest ET (of >1000 mm year^−1^) occurred in regions where mean precipitation (P) exceeded 2500 mm year^−1^, while it was lowest (<200 mm year^−1^) in areas with a lower annual P (<50 mm year^−1^). On the other hand, we observed distinct spatial patterns in ET in the interior of the Ethiopian and Kenyan Highlands (in the HoA region) and at locations (15° S) with historical ET values of 600–800 mm year^−1^.

Figure 4b–e shows the projection of the spatial distribution of the multi-year mean ET obtained by subtracting the historical mean from the entire time series. Under all SSP-RCPs scenarios, the ranges of the spatially averaged ET changed in overlap but were different in relative magnitudes. In general, under all four SSPs-RCPs, decreases in ET (Figure 4b–e) were projected mainly over geographical locations located 18° S–30° S. We observed spatial patterns of high magnitudes (>100 mm year^−1^) similarly distributed in the arid regions of the Kalahari Desert.

The spatial pattern of slight increases in ET under SSP1-2.6 and SSP2-4.5 was similarly distributed in regions north of the equator. For example, the spatial pattern of high magnitudes of projected values (i.e., <50 mm year^−1^) in ET under SSP1-2.6 and SSP2-4.5 was prominent in small pockets at 20° N–35° N (in the Sahara Desert), patches around 05° N–equator, i.e., the Guinean coast), the equatorial region (in the Congo Basin), and patches in the HoA region (see Figure 4b,c). Additionally, patches of high values of <50 mm year^−1^ occurred at the tips of the HoA region and in the peripheral regions of bodies of open water.

Spatially, the geographical distribution of the projected increase in ET was similarly distributed in the same regions as shown in Figure 4d,e. However, the pronounced pattern of increases in the projected ET was detected under SSP3-7.0 and SSP5-8.5. We observed high values, increasing up to >100 mm year^−1^, in patches located at the northeast tips of Mozambique (at locations around 15° S). In addition, we observed an interesting but complex spatial pattern of high values of the projected ET (>150 mm year^−1^) at locations around 15° S (the northeast tips of Mozambique) and in the peripheral regions of large bodies of open water.

**Figure 5:**
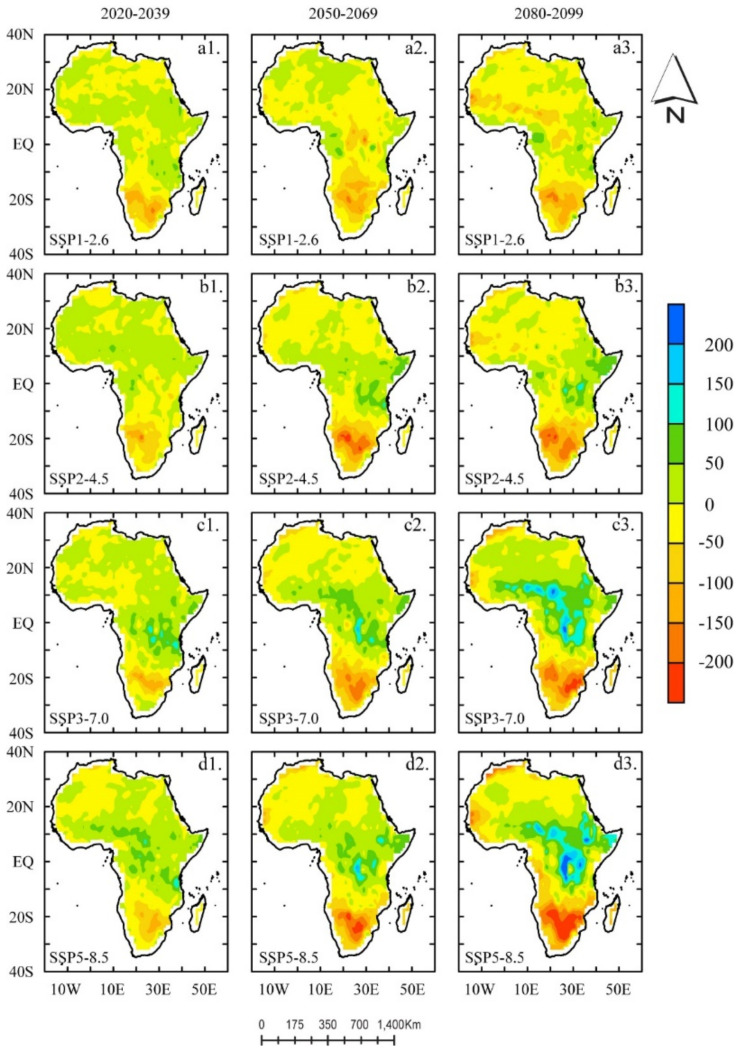
Spatial distribution of multi-year mean ET based on the historical mean (1995–2014) under the SSP1-2.6 (**a1**–**a3**), 2−4.5 (**b1**–**b3**), 3−7.0 (**c1**–**c3**), and 5−8.5 (**d1**–**d3**) scenarios in three future periods (2020–2039; 2020–2069; and 2080–2099). A positive (negative) ET value denotes a projected increase (decrease). The unit is mm year^−1^.

To design short- to medium-term policies to mitigate the potential impacts in different sectors of society, it crucial to analyze and understand the changes in the lengths and timing of the ET within shorter future timeframes. Here, we present an analysis of three different future periods: near future (NF) (2020–2039), mid-century (MC) (2050–2069), and end-of-the-century (EC) (2080–2099), for all SSPs to provide a better understanding of their seasonality and quantify their relative spatial distribution of ET in future periods. Figure 5 illustrates the spatial distribution of the multi-year mean ET for three different future periods for the SSPs. From the figure, positive (negative) ET values denote a projected increase (decrease).

In general, under all SSPs and periods, we observed similarities in the spatial distribution of projected increase (decrease) of ET in the same geographical regions. For each period, we observed a slight exception in regions around 15° S–18° S (with a pronounced pattern of ET detected); this showed similar magnitudes of positive (negative) ET values irrespective of the SSPs under consideration. Spatially, we detected a pronounced pattern of positive (negative) ET at MC (Figure 5c2,d2) and EC (Figure 5c3,d3) south of the equator, namely, 18° S–15° S (30° S–18° S), respectively. Under all four SSPs, SSP1-2.6 and SSP2-4.6 showed a similar spatial pattern of ET compared to the SSP3-7.0 and SSP5-8.0 scenarios. However, CNRM-CM6 simulated a pronounced pattern of ET in SSP3-7.0 (c1, c2, c3) and SSP5-8.0 (d1, d2, d3) in comparison to the SSP1-2.6 (a1, a2, a3) and SSP2-4.6 (b1, b2, b3) scenarios.

### 3.2. Linear Trend in Projected ET

**Figure 6:**
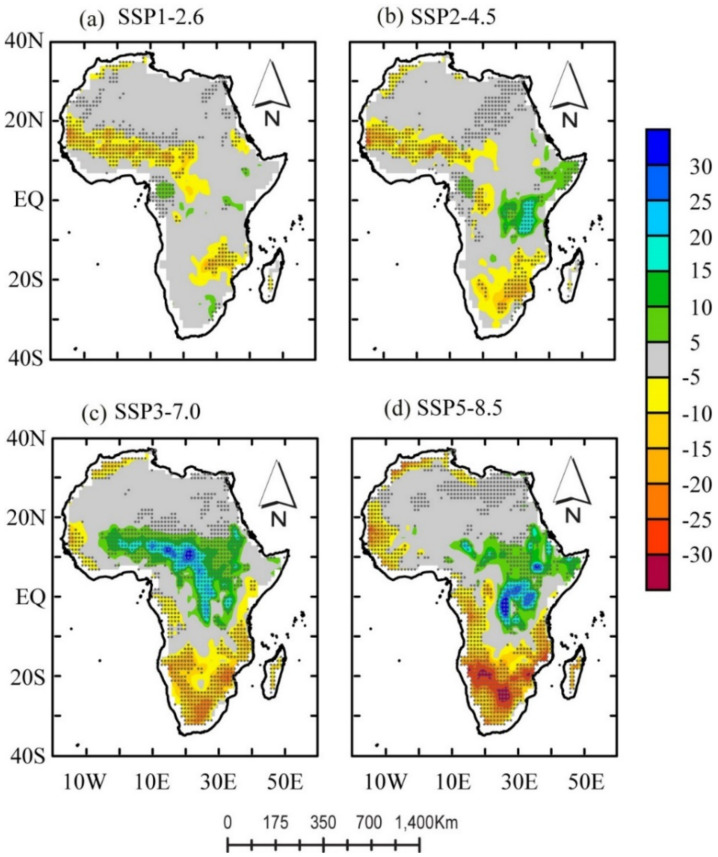
The linear trends of evapotranspiration under the SSP1-2.6, 2-4.5, 3-7.0, and 5-8.5 scenarios in the future period (2020–2099). The positive values depict an increase, and the negative values depict a decrease. The unit is mm decade^−1^. The black dots indicate that the trend passes the 0.05 significance test.

To detect changes, we conducted a linear trend analysis based on the Mann–Kendall test and Sen’s slope test [65,66,67]. We calculated the trends on a per-pixel basis. The trends were tested for significance, and the shaded areas represent grid points in which the linear trends are significant at a 95% confidence level. Figure 6 depicts the spatial pattern of the CNRM-CM6-projected linear trends for future climate (2020–2099) under all SSP-RCPs. The positive values depict an increase, and the negative values depict a decrease.

Generally, as seen in Figure 6, the projected changes in ET were diverse in the projected spatial distribution. We observed significant differences in their relative magnitudes (and direction) of linear trend values under all SSPs. Under the SSPs, we observed locations at 15° S–30° S (the Kalahari Desert, extending to the tips of Mozambique) presenting an interesting picture. For example, we observed locations south of the equator (i.e., at 15° S–30° S) showing increasing dryness as the emission scenarios increased from low to high.

A strong increase in projected dryness conditions occurred under the SSP3-7.0 and 5-8.5 scenarios, whereas relatively less dryness or no significant trend was found north of the equator (20° N–30° N, Sahara Desert) under the high-emission scenarios (SSP3-7.0 and 5-8.5). For the same geographic locations (i.e., 20° N–30° N and 15° S–30° S), we observed less dryness or no significant trend under SSP1-2.6 and SSP2-4.6.

In Figure 6a–d, we detected a striking bipolar linear trend in the semi-arid region (west of the Sahelian belt) and the equatorial region (in the Congo Basin). For example, SSP1-2.6 and SSP2-4.5 showed projected dryness trends, whereas SSP3-7.0 and 5-8.5 showed wetness trends. A spatially distinct pattern of negative projected ET trends ranged from 5 to 15 mm. In contrast, SSP3-7.0 and SSP 5-8.5 projected relatively high values in magnitudes of >5–30 mm/10 a. The changes in trends (Figure 6a,b) were similar in the Sudano-Sahel belt and equatorial regions. We observed projected dryness (wetness) conditions under SSP1-2.6 and SSP2-4.5 (SSP3-7.0 and SSP5-8.5). These results suggest that future dryness (wetness) conditions will most likely increase dramatically in these regions, especially under SSP1-2.6 and SSP2-4.5 (SSP3-7.0 and SSP5-8.5).

**Figure 7:**
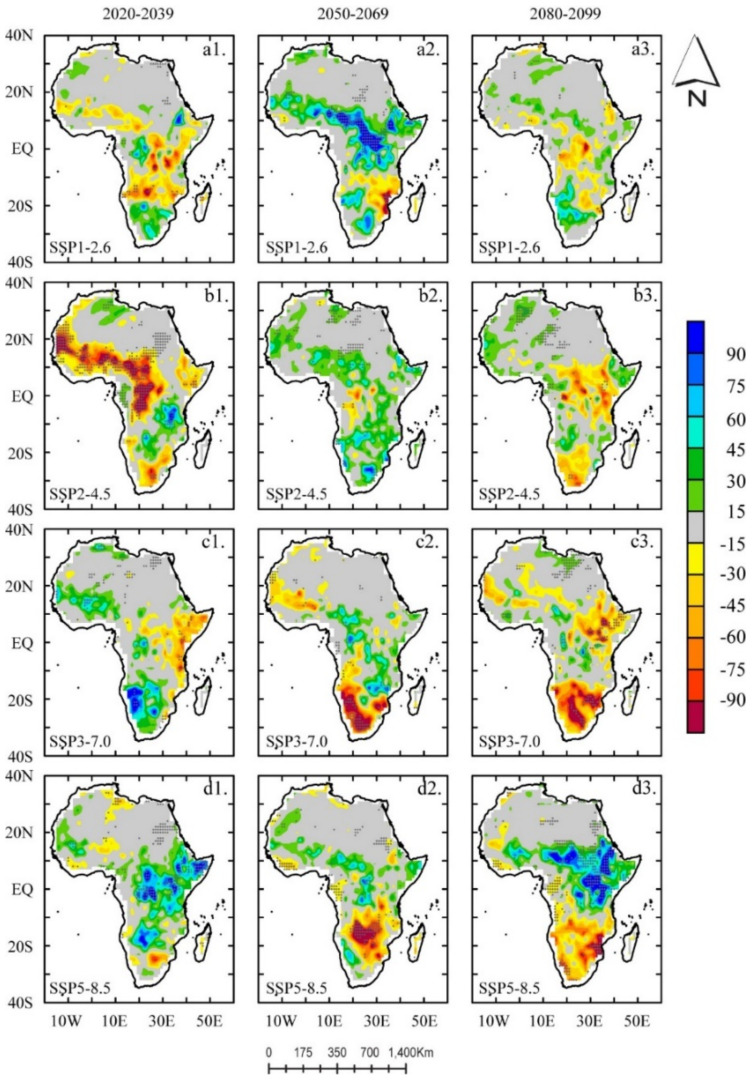
The linear trends of evapotranspiration under the SSP1-2.6 (**a1**–**a3**), 2-4.5 (**b1**–**b3**), 3-7.0 (**c1**–**c3**), and 5-8.5 (**d1**–**d3**) scenarios in three future periods (2020–2039, 2020–2069, and 2080–2099). The unit is mm decade^−1^. The black dots indicate that the trend passes the 0.05 significance test.

We extended the analysis to understand how the trends (both magnitude and direction) under three different shorter future periods may improve our understanding of their seasonality and help quantify their relative spatial distribution of ET in future periods. In Figure 7, we present the linear trends of ET in three future periods: NF, MC, and EC, under the same radiative forcing. Generally, from the figure, positive (negative) ET values represent a projected wetting (drying) trend per decade.

From Figure 7, we observed that there was spatial heterogeneity in the trend results with differences in their relative magnitudes for all three periods under all radiative forcing.

In the NF, we observed the transition zones showing a drying trend of about 5–15 mm/10 a under the SSP1-2.6 and SSP2-4.5 scenarios in the Sudano-Sahel–Guinean coast belt towards the equatorial zones in the Congo Basin and regions with distinctly open water bodies and channels (in HoA). However, we observed a more distinct and sharply decreasing trend under SSP2-4.5 (Figure 7b1) than SSP1-2.6. Then, in the MC, the trend changed in magnitude and direction. A strong wetting trend was observed in the MC, but this time SSP1-2.6 produced more wet scenarios than SSP2-4.5 (Figure 7a2,b2) with ET values ranging from <15 to >30 mm/10 a, mainly in semi-arid regions and equatorial zones of Africa.

Finally, EC projections (Figure 7a3,b3) showed a similar spatial distribution of ET trends values to the NF (Figure 7a1,b1), particularly in the HoA regions extending to the Kenyan Highlands, and areas north of Lake Victoria to the southeast tip of Mozambique (i.e., 18° S–22° S) under SSP1-2.6 and SSP2-4.6. Distinctively, we observed that the spatial distribution of the linear trends of ET displayed a bipolar result in depicting linear trends (in both magnitude and direction) in different geographical locations over the study region.

Under SSP1-2.6 (SSP2-4.6), we observed a wetting (drying) trend in patches surrounding the Kalahari Desert extending to large patches at 18° S–25° S (in the southwestern portion of Southern Africa) for NF and EC. Similarly, in the SSP3-7.0 and 5-8.5 scenarios, we observed a bipolar result in depicting linear trends (in both magnitude and direction), particularly in the HoA extending to the southeast tip of Mozambique (i.e., 18° S–22° S). However, patches surrounding the Kalahari Desert extending to large patches at 18° S–25° S (in the southwestern portion of Southern Africa) showed similar trends (in magnitude and direction). Here, we observed that a trend (in 2020–2039) would eventually shift to a wetting trend (in 2040–2069) and return to a drying trend (at end-of-the-century) at the rate of <15 to >30 mm/10 a. Interestingly, we noted that under SSP5-8.5, a significantly wetting trend over the 21st century was observed over the HoA, except for slight wetting shown in MC.

## 4. Discussion

Understanding past drivers of climate change is crucial for assessing future climate change. The analysis of the CNRM-CM6 model captures well the annual (Figure 2) and seasonal (Figure 3) cycle of ET across Africa. We found that the decreasing trends of annual mean ET across the continent under all SSP-RCPs (Figure 2) was consistent with the T and P seasonality over the region from CMIP6 [27], as in response to changes in P and T, ET is naturally expected to change across the region. Interestingly, our findings demonstrated that SSP3-7.0 and SSP5-8.5 showed a higher rate of changes than the other two scenarios. Other studies found similar results for higher emissions [41,71,72]. For example, Almazroui et al. [27] found a large heterogeneity of T associated with larger radiation forcing in similar regions. This may be attributed partially to the larger uncertainty of the changes in T associated with larger radiation forcing [64]. The findings for the seasonal cycle for the historical period were unimodal and close to the cycle south of the equator. This result is consistent with the CMIP6 P seasonality found in Southern Africa by [73]. However, the future climate presented an opposite seasonal cycle for all the SSP-RCPs (Figure 3). This is not surprising due to the different seasonal cycles in the north and south of the equator; the changes in seasonal mean may result in enhanced seasonality.

The dry seasons (i.e., winter and spring) experienced higher monthly ET values, while the wet seasons (i.e., summer and autumn) experienced lower monthly ET values (Figure 3). The ET seasonality coincided with and followed unimodal P patterns south of the equator. Conversely, we projected a trimodal ET pattern for SSP-RCPs, consistent with the P patterns in the equatorial regions. This may be linked to the increase in projected P shown in the CMIP6 ensemble [27]. The relative change in future ET may suggest a wet condition in OND, JJA, and in January–February (J–F). Higher emissions (SSP3-7.0 and SSP5-8.5) projected greater wetting conditions than the other two scenarios.

The spatial patterns of ET for the historical and future climate were examined to provide a better understanding of their seasonality and quantify their relative spatial distribution in the future period (2020–2099) across the continent. We noted that the simulated ET amounts were maximum over the equatorial regions (between 5° S and 5° N); this is consistent with the P seasonality in West Africa [74,75] and the equatorial region [27,76]. The amount reduced moving towards the humid–tropical region, the Sahelian region, and, finally, in the arid region of the Sahara Desert (Figure 4a). The patches of arid conditions found in the HoA had a more pronounced decrease in ET than in the Kalahari Desert (in the south). The humid–tropical zones (along with the Guinean coast) and the interior of the Cameroonian, Kenyan, and Ethiopian Highlands provided a comparatively similar mean ET. The ET pattern recorded is consistent with CMIP6 P patterns found in East Africa [77], North Africa [78] and Southern Africa [73].

Compared to the spatial patterns under high emissions, SSP3-7.0 and SSP5-8.5 showed simulated ET values relatively similar to the highest ET values, which were located over the equatorial regions, with values increasing from the Congo Forest to the HoA (Figure 4d,e). These results for the SSP3-7.0 and SS5-8.5 scenarios show that the humid–tropical and equatorial zones are likely to experience a significant change in ET, especially in Central and Eastern Africa; however, the same cannot be said about SSP1-2.6 and SSP2-4.5, as most regions are predicted to experience a low amount of ET. Almazroui et al. [27] projected high (low) P in similar locations. These findings are not surprising as it may be expected that an increase in T would increase P and ET and thus intensify the hydrological cycle. The regions with a larger projected annual mean P to global warming may likely have more ET, suggesting that more P (wet conditions) under higher emission scenarios have a wetter pattern. This is consistent with the higher P amounts projected for the regions (see [27,37]) in the CMIP6 models. The findings of the spatial pattern of ET for the historical and future climate examined here provide our understanding of their seasonality and quantify their relative spatial distribution of ET in the future period (2020–2099) across the continent.

The results of the trend analysis (Figure 6) show that the projected changes in ET are diverse, with regions in the humid–tropical and equatorial zones, especially in Central and Eastern Africa, having significantly larger trend magnitudes under all SSPs. Regions located in the Mediterranean (located in the northern tip of Africa) showed significantly decreasing trends. This result is consistent with P [78] and T [27,76] trends over the north of Africa. The results of the linear trend values for SSP1-2.6 and SSP2-4.5 are close to each other and the same was found for higher emissions (SSP3-7.0 and SSP5-8.5). The difference between SSP3-7.0 and SSP5-8.5 shows that SSP5-8.5 had more pronounced signals and covered a much larger area (Figure 3).

Africa’s agriculture system is largely rainfed, and the growing seasons follow the P patterns in space and time. Thus, from an agriculture policy perspective, these results suggest that wet (dry) conditions would likely be favorable (unfavorable) for farming in regions that coincide with the start of the planting seasons in water-limited regions. On the other hand, from an urban policy perspective, regions located in wetter climate conditions may likely experience increased P and subsequently an increased risk of flooding. Conversely, dry conditions in the other months indicate that drier conditions may experience low or no P, resulting in an increased drought risk [9]. The African region is the most vulnerable to climate change impacts and arguably has a weak system to facilitate evidence-based policy implementation designed for adaptive and mitigative strategies. This study highlighted a key climate variable (i.e., the ET) from one CNRM-CM6 dataset. From the perspective of public health, ET change and variability in the region in future scenarios may have implications for the transmission of diseases, such as malaria, in some parts of the region, consistent with [24,27,79,80] findings, but for P and T trends and variability.

We demonstrated the temporal pattern and spatial distribution of simulated ET in the various climate regions of Africa using the CNRM-CM6 under all SSP-RCPs. Detecting climate changes is crucial in developing adaptation and mitigation measures at the regional and local scales. Our future dependence will be further influenced by increased evaporative demand by the atmosphere. Particularly, these changes imply future water availability for use by the different sectors of the society that depend on it. However, readers are advised to use these results with caution as further studies using multi-model methods (as they become available) may show which models perform well in which climate regions, thus providing more information on tailor-made mitigation and adaptive measures for different climate conditions.

## 5. Summary and Conclusions

Ongoing global warming has brought the discussion of mitigating the adverse impact of extreme events to the forefront. We focused on ET, as future changes are projected to influence evaporative demand by the atmosphere and potentially impact future water availability for use by societies and the ecosystems.

According to Eyring et al. [12], CMIP6 has improved spatial resolution, and the physics (concerning parameterization and components) have also significantly improved. Additionally, the CMIP6 projections include socioeconomic impacts on the climate, making them more realistic and usable. In recent times, much of the literature has investigated projections of future climate in different regions worldwide for a wide variety of climate variables, but without ET. This study aimed to contribute to the increasing demand for scientific work by the climate science community by investigating the projection of simulated ET using CMIP6 models.

We used CMIP6 CNRM-CM6 to assess future changes of the simulated ET from a regional perspective and to examine the long-term changes (trends) across Africa for both historical (1995–2014) and future SSP-RCPs scenarios (i.e., SSP1-2.6, SSP2-4.5, SSP3-7.0, and SSP5-8.5). The choice was based on data availability at the time of this study.

The CNRM-CM6 was able to reproduce the projected ET for future climates by quantifying ET seasonality across continental Africa in both space and time. CNRM-CM6 captured the distribution of historical and projected ET over Africa at both annual and seasonal scales. Historical analysis showed a striking distinct spatial pattern of average annual ET across diverse climatic zones. The arid regions (the Sahara Desert and the Kalahari areas) indicated declining ET trends by the end of the 21st century, with larger values under the SSP5-8.5 scenario than under the SSP1-2.5 scenario. We observed a dominant pattern of high (low) ET values projected in equatorial and tropical–humid regions. The equatorial and tropical–humid regions shown demonstrated a projected wetting trend in all four SSPs-RCPs. The highest trend values were in the equatorial region and under high emissions (i.e., SSP3-7.0 and SSP5-8.5). This study thus provides an insight into Africa’s changing climate in the 20th and 21st centuries.

In general, we suggest the need for modelers and forecasters to pay more attention to how the simulated ET changes will impact the generation of extreme events. The findings of this study provide useful information for water resources managers to develop specific measures to mitigate extreme events in the regions most affected by climate variability. However, these results are based on one GCM and therefore should be treated with caution. Further research using multi-model ensembles (as the data become available) and possible keys drivers over the region may provide additional information on ET climatology and trends over Africa.

## Data Availability

The data presented in this study are available on request from the corresponding author.
